# On Two Not Generally Known Pursuits of the United States Government in the Interest of Practitioners of Medicine and Zoologists*Read by invitation at the twelfth semi-annual meeting of the Eighth District Medical Association, at Bay City, Texas, October 27, 1910.

**Published:** 1911-01

**Authors:** Charles A. Pfender

**Affiliations:** Assistant, Children’s Diseases, Central Dispensary and Emergency Hospital, Washington, D. C.


For Texas Medical Journal.

### On Two Not Generally Known Pursuits of the United States Government in the Interest of Practitioners of Medicine and Zoologists.*

BY CHARLES A. PFENDER, M. D., ASSISTANT, CHILDREN'S DISEASES, CENTRAL DISPENSARY AND EMERGENCY HOSPITAL, WASHINGTON, D. C.

*Mr. President, Ladies and Gentlemen:*

When requested by your most efficient Secretary to present a paper before this meeting of the Eighth District Medical Association of the Lone Star State, I was truly at a loss what topic to select. It has always been a pleasure for me to give heed to the reports of the achievements of others in the pursuits of their avocation and, perhaps, to indulge in the discussion of subjects of scientific and practical interest to all earnest and faithful practitioners of medicine. It occurred to me that a number of good papers dealing with the clinical and pathological phases of our work would be presented on this occasion, and this thought engendered another, namely, that the presentation of a few facts concerning some not generally known agencies which have made possible some of the epochal triumphs in the realm of medicine might prove of timely interest to those who convene here today. I, therefore, welcome this opportunity to place before you a brief outline of the most important points.

Some of the more fortunate ones assembled here have been granted the privilege of personal investigation, and to those a repetition of familiar themes will, no doubt, recall pleasant and profitable hours, while those to whom the goddess of fortune has been less generous may gain a bird's-eye view, one might say, of the catacombs of the thoughts of men who have in many instances long since passed away.

Every true American citizen shares the just pride of national achievment. As physicians, we may take special delight in the fact that we have in our midst a National Medical Library without peer in the western hemisphere and superior by far to most of the medical libraries of the Old World. It is marvelous to contemplate the rapid growth of this institution. Something more than a generation ago Surgeon General Lovell of the United

*Read by invitation at the twelfth semi-annual meeting of the Eighth District Medical Association, at Bay City, Texas, October 27, 1910.

States army began to collect medical books and journals for the use of the army surgeons connected with the Surgeon General's Office of the War Department. For the first thirty years, progress was very slow and difficult. The earliest accurate printed statement that I have been enabled to find and which gives an exact status of the condition of affairs during the earlier years in the history of this library is contained in the first published catalogue of the library, which appeared May 10, 1864. This pamphlet volunteers the information that the library comprised about 1800 volumes at that time. This catalogue is a very modest tome. It is composed of only twenty-seven leaves, and is considerably less pretentious than an ordinary prescription pad. What a marked contrast with the achievement of today! But of that later.

Shortly after the close of the war—in the fall of 1865—Dr. John S. Billings, after a splendid career as army surgeon, was appointed to take charge of the Medical Library of the office of the Surgeon General. Subsequent events confirmed the wisdom of the selection. He quickly realized the possibilities of the position, and so strenuously applied his knowledge and learning in acquiring additional medical works through congressional appropriation and by personal appeal to physicians throughout our land that soon the institution had risen from an inferior station to one that merited and received widespread recognition. Dr. Billings is in deed and in truth the creator of this treasury of information which stands today a glowing testimonial to his efforts. The medical profession owes much to this eminent scholar and physician, and it is largely through his energy that the library now ranks as the third of the large medical libraries of the world in point of number of volumes, for it is equaled only by the Library of the Faculty of Medicine of Paris, France, which contains 200,000 volumes, and the Library of the Imperial Academy of Medicine of St. Petersburg, Russia, which is composed of 170,000 volumes. In the beginning of 1909, our library contained 168,879 volumes and 297,560 pamphlets, almost half a million of medical works, including many of the choicest treasures of medical literature.

Its scope of usefulness has no limitations, for from the wide shores of the great Atlantic to the broad expanse of the proud Pacific, from the bleak heights of grim Alaska to the placid gulf of the Sunny South come daily appeals from representatives of the medical profession for information contained in this great fund of medical knowledge. The fame of the library has spread beyond the bounds of our own domain and from other climes and lands beyond the seas medical men of note come to consult at this Mecca of scientific literature work of medicine *nowhere else obtainable,* and thus they pay an eloquent tribute to the value of the collection. To the words of the assistant librarian, Dr. Fielding H. Garrison, that the library contains "the most unrivaled collection of medical periodicals in the world, is singularly rich in incunabula and the works of earlier writers," we might add that modern literature is constantly being supplied through agents at home and abroad, thus keeping abreast with the never-ceasing progress of the day.

Not only is the library replete with volumes of ancient, medieval and modern literature, but it also contains an author and a subject index catalogue whose magnitude must astound the uninitiated. This idea had its conception in the massive brain of Dr. Billings, and in 1873 work was actually begun. Dr. Robert Fletcher was appointed principal assistant in the preparation and printing of the Index-Catalogue of the Library of the Surgeon General's Office of the United States Army, and, in 1880, the first volume, containing 888 pages, appeared. Subsequent volumes followed in quick succession as the work progressed, and the first series, from A to Z, comprising 16 volumes in all, was completed in 1895. As the accumulation of medical literature made a second series necessary, this was begun at once, and to date 14 more volumes, from A to R, inclusive, have appeared. The remainder is being prepared for the printer with the greatest possiple dispatch. These thirty printed volumes contain on an average of 1000 pages each, quarto size, the entire set weighing in the neighborhood of 245 pounds. I wish I could show you the contrast between the little author's catalogue of 1864, which weighed only a few ounces and contained twenty-seven leaves, and this magnificent work of 30,000 pages or more.

A brief summary of contents will in a measure demonstrate the immense scope of the index work collaborated by the faithful corps of library workers in the Surgeon General's Office.

AUTHOR TITLES.

| | Titles. | Volumes. | Pamphlets. |
|---|---|---|---|
| Vols. 1-16 (1880-1895) ....... | 176,364 | 85,663 | 151,504 |
| 2 s., v. 1-14 (1896-July 1, 1910) | 130,073 | 60,336 | 125,105 |
| Total ............... | 306,437 | 145,999 | 276,609 |

SUBJECT TITLES.

| | Booklets. | Jour. Arts. | Portraits. |
|---|---|---|---|
| Vols. 1-16 (1880-1895) | 168,557 | 511,112 | 4,335 |
| 2 s., v. 1-14 (1896-July 1, 1910) | 110,190 | 442,780 | 677 |
| Total | 278,747 | 953,892 | 5,012 |

It is well worth remembering that this catalogue is the *only printed work of its kind in the world* today, and is used by medical men throughout Europe and America. It consists of a full catalogue of the works contained in the library and a complete bibliography of the works of the literature of medicine. Fifteen hundred copies are printed, and these are judiciously distributed among the universities, libraries and medical institutions of the United States and foreign countries, enabling workers in scientific medicine to pursue their research with systematic precision and with the greatest possible dispatch. There may be a few medical libraries that contain more books than the Library of the Surgeon General of the Army, but the latter excels in point of utility by virtue of its splendid appointments.

Not content with their success in the above mentioned undertaking, Dr. Billings and Dr. Fletcher extended their activities in the conception of a *monthly bibliography of current medical literature,* and to this work Dr. Fletcher in particular has devoted much time, energy and means. This work is probably known to most of you as the Index Medicus. At the end of each volume is appended an annual index, which covers the medical literature for the year. The vast amount of work entailed by the preparation of such a bibliography can be fully appreciated by those only who are conversant with an undertaking of this nature, and the members of the library force entrusted with this responsibility deserve great credit for the exceptional ability they have shown in its arrangement.

After the Index Medicus had been under way for a number of years the financial horizon grew dark and threatening; the whole enterprise seemed doomed to shipwreck, when, in 1903, the Carnegie Institution, realizing the necessity for the continuation of this publication, assumed the financial obligations and thus happily insured the permanency of its production. It is more than gratifying to note in this connection that, in acknowledgment of his masterful work, Dr. Fletcher was recently awarded a medal by the Royal College of Physicians of London.

We willingly admit that liberal support through appropriations made by Congress served greatly in furthering the phenomenal growth of the library, but it is also true that quite a large number of books in the library have been contributed from time to time by medical and other scientific men in this country who had the welfare of the library at heart. Contributions of this nature supplemented by purchase have filled many gaps and perfected series that were long incomplete, and thus enhanced the value of the collection.

A goodly number of reprints of articles published in the medical journals have also been sent in by the authors thereof, and it seems to me this should be encouraged. The donation of a reprint pre-empts for it a place upon the shelves of the institution and in addition insures a prompt and correct index of the article. When it is borne in mind that now and then a journal number in which the article may have appeared is lost either in transit through the mails or otherwise, we can appreciate how the donation of such a reprint may save the work from probably partial oblivion anyway, and in rare cases it may serve to establish priority claimed by the writer.

The printing of the Index-Catalogue is provided for by an annual appropriation by Congress of $10,000. Inimical interests have arisen from time to time and have attempted to cut off this appropriation, but the appreciative attitude of the leaders of the medical profession of the United States has thus far always been successful in defeating the objection raised.

The Library of the Surgeon General's Office is not a public library in the strict sense of the word, as it is primarily intended for the use of the Medical Corps of the Army. An arrangement exists, however, by which physicians may obtain books on payment of a suitable deposit to insure their return or to protect against loss by fire, negligence or otherwise. Physicians residing in other cities may have books sent to them by application through universities, public libraries or medical societies in their vicinity, which thus assume the responsibility for their safe return. Those desirous of perusing rare works are always privileged to visit the library and view them there. It would be unwise, to say the least, to allow these books to leave the institution, as many could not be replaced in case of loss.

An immediate adjunct to the main library hall is the reading room, which is open to medical men who may here pursue their research undisturbed from 9 a. m. to 4 p. m. every day of the week, except Sunday. This room is equipped with copies of the

Index-Catalogue, Index Medicus, and other works of reference, and is in charge of a competent assistant librarian.

Since the time Dr. Billings terminated his connection with the library in 1895, this institution has been under the supervision of medical officers detailed from the Army Medical Corps, and these men have advanced the work so nobly begun by their predecessor. The present incumbent, Lieutenant Colonel Walter D. McCaw, deserves special mention for the great interest he has manifested in the furtherance of the work by the introduction of modern progressive methods which have placed the library second to none in the world. Dr. Robert Fletcher, now in his 88th year, and approaching the termination of a long and faithful career, is still directing the bibliographic work with characteristic zeal and vigor in spite of his years, and in this he is ably seconded by his principal assistant, Dr. Fielding H. Garrison, who is also associate editor of the Index Medicus.

Ladies and gentlemen, I have sketched for you the most important facts relating to this institution, and would bespeak your kind attention for but a few moments longer in order that I may quite briefly touch upon another great work under the auspices of the government which is being carried on by the Zoological Divisions of the Department of Agriculture and the Public Health and Marine Hospital Service. I refer to the Index-Catalogue of Medical and Veterinary Zoology. The Index-Catalogue of the Library of the Surgeon General deals with the subjects of interest to the medical practitioner and scientist in general, while the Index-Catalogue of the Zoological Divisions is devoted entirely to medical zoology. It is an exclusive publication, the only one of its kind in existence today, and the hope expressed by the chief of the Bureau of Animal Industry "that this catalogue would prove of very great value to persons interested in zoology in its practical relation to human and veterinary medicine and public hygiene" has met with entire fulfillment, if we may accept the demand for copies and the laudatory expressions of specialists in this field on both continents as proof sufficient for our statement.

The inception of this catalogue may be traced to the ever virile minds of Prof. Charles Wardell Stiles and his faithful assistant, Dr. Albert Hassall. For years it remained as an unpublished card catalogue, constant additions being made by the authors and their limited corps of assistants, until, comparatively speaking, it attained stupendous proportions. The universal need of publishing this material became more and more apparent as others engaged in similar lines of work realized the value of this collection and began to urge its publication so that it might be accessible to them also. It was, therefore, decided to print it in three sections, namely, as an Author Index, a Subject Index and a Host Index. The Author Index appears in an edition of 2568 copies as Bulletin No. 39, Bureau of Animal Industry, with continuous pagination, in parts not exceeding 100 pages each, and the parts are published as soon as edited. Part I, A to Azevedo, was published in 1902, and other parts have appeared from time to time. The last number, comprising the letters X, Y and Z, is at present in the hands of the printer, and when this appears the first series will have been completed. The greatly augmented interest in zoological fields recently manifested by physicians and others not necessarily zoologists has led to such a rapid growth in medico-zoological literature that the unpublished cards of the indexed material have already attained proportions sufficient to suggest the advisability of printing a second series.

The arrangement of the journal references of the Author Index is so decidedly original, simple and complete as to invite a brief outline of the method in vogue. In every case an effort is made to obtain every possible clue that may aid in the identification of a reference. Beginning with the author's full name, title, place of residence, date of birth and death, biographic and bibliographic references and cross-references to other writers, we have the date of publication of the article, the title of the article, the title of the journal in which it appears and the place of publication of the latter, in turn designation of the section, series, volume, number, month of the year, pages, plates, figures, date of manuscript, and last, but not least, a notation of the libraries in which the work may be found.

For example:

Braun, Max[imilian]. [Prof. Zoology, Königsberg i. Pr.] [1850—.]

1901 e.—Zur Kenntniss der Trematoden der Säugethiere. Zool. Jahrb., Jena, Abt. f. Syst., v. 14 (4), 18. März, pp. 311-348, pls. 19-20, figs. 1-17. [MS. dated August, 1900.] [Wa, Ws].

An effort is also made to arrange the articles chronologically as they appear each year and to give each article an alphabetical designation which explains, in this instance, the letter (e) following the date of publication. The designations Wa and Ws signify the libraries of the Department of Agriculture (Washington Agriculture) and the Smithsonian Institute (Washington Smithsonian) of Washington, D. C. It must not be overlooked that abstracts as well as the original articles are indexed so that in cases where the original article is not available to the reader reference to the abstracts may serve his purpose. This has been found of great value, particularly in such cases as Russian original articles, an abstract in some more modern language frequently sufficing the needs of the investigator.

With regard to the distribution of this publication, the notice prefacing each part reads that "this bulletin is not for general free distribution, but is intended for use of libraries, educational institutions, experiment stations, laboratories, sanitary officials, and investigators. Persons desiring private copies can obtain them by purchase from the Superintendent of Documents, Government Printing Office, Washington, D. C., or by exchange with the Bureau of Animal Industry. In ordering copies, state distinctly which number is desired."

The subject catalogue considers some groups more completely than others. This is due to the fact that the catalogue is simply the working index prepared for the convenience of the Zoological Laboratory. The Cestodes, Trematodes, and thorn-headed worms will be more complete than the Protozoa, sponges, poisonous snakes, etc. The list of Trematoda and Trematode Diseases was published in June, 1908, as Bulletin No. 37 of the Hygienic Laboratory of the United States Public Health and Marine Hospital Service, and contains 401 pages. The Cestodes are now in press, while the work is being pushed on the Nematodes, etc. These will be published as soon as they can be edited.

The Host Card Catalogue is growing rapidly and will probably follow the other publications. As the force engaged in the preparation of this material is quite small, it will be some time before the latter will appear.

I feel that I am beginning to trespass on your good nature. To enter into further detail at this time would only weary you. I would not care to leave with you impressions akin to those expressed on one occasion by a buxom colored lady who had applied to a magistrate for a divorce. After considerable parley, during which the dusky dame failed to furnish any reasonable grounds for procedure, the irate judge finally demanded, "Why in the name of heaven are you asking for a divorce then?" In reply she said, "Your Honor, I just sort o' lost my taste for him."

Plain facts are always more or less tiresome, more often more than less so, and I will close my remarks with the hope that upon your next visit to the Capital City you will avail yourself of the opportunity of visiting the institution I have spoken of and obtain a more comprehensive and correct idea of the working of these projects at close range, for, no matter how well described a subject may be, personal observation intelligently conducted ranks paramount.

